# Antigen Delivery to DEC205^+^ Dendritic Cells Induces Immunological Memory and Protective Therapeutic Effects against HPV-Associated Tumors at Different Anatomical Sites

**DOI:** 10.7150/ijbs.57038

**Published:** 2021-07-13

**Authors:** Mariângela O. Silva, Bianca S. Almeida, Natiely S. Sales, Mariana O. Diniz, Luana R.M.M. Aps, Karine B. Rodrigues, Jamile R. Silva, Ana C. R. Moreno, Bruna F.M.M. Porchia, Fernando B. Sulczewski, Silvia B. Boscardin, Luís C. S. Ferreira

**Affiliations:** 1 Vaccine Development Laboratory, Department of Microbiology, Institute of Biomedical Sciences, University of São Paulo, São Paulo, SP, Brazil; 2 Laboratory of Antigen Targeting to Dendritic Cells, Department of Parasitology, Institute of Biomedical Sciences University of São Paulo, São Paulo, Brazil

**Keywords:** DEC205 receptor, cancer immunotherapy, dendritic cells, HPV

## Abstract

The generation of successful anticancer vaccines relies on the ability to induce efficient and long-lasting immune responses to tumor antigens. In this scenario, dendritic cells (DCs) are essential cellular components in the generation of antitumor immune responses. Thus, delivery of tumor antigens to specific DC populations represents a promising approach to enhance the efficiency of antitumor immunotherapies. In the present study, we employed antibody-antigen conjugates targeting a specific DC C-type lectin receptor. For that purpose, we genetically fused the anti-DEC205 monoclonal antibody to the type 16 human papillomavirus (HPV-16) E7 oncoprotein to create a therapeutic vaccine to treat HPV-associated tumors in syngeneic mouse tumor models. The therapeutic efficacy of the αDEC205-E7 mAb was investigated in three distinct anatomical tumor models (subcutaneous, lingual and intravaginal). The immunization regimen comprised two doses of the αDEC205-E7 mAb coadministered with a DC maturation stimulus (Polyinosinic:polycytidylic acid, poly (I:C)) as an adjuvant. The combined immunotherapy produced robust antitumor effects on both the subcutaneous and orthotopic tumor models, stimulating rapid tumor regression and long-term survival. These outcomes were related to the activation of tumor antigen-specific CD8^+^ T cells in both systemic compartments and lymphoid tissues. The αDEC205-E7 antibody plus poly (I:C) administration induced long-lasting immunity and controlled tumor relapses. Our results highlight that the delivery of HPV tumor antigens to DCs, particularly via the DEC205 surface receptor, is a promising therapeutic approach, providing new opportunities for the development of alternative immunotherapies for patients with HPV-associated tumors at different anatomical sites.

## Introduction

The immune system has long been studied as a key element in the fight against cancer. Years of research have been devoted to trying to understand how to modify the immune system for the benefit of patients. Currently, the use of immunotherapy represents a pivotal advance in oncology through different strategies that stimulate and amplify the immune response, promoting the elimination of tumor cells 1,2. In this landscape, the activation of dendritic cells (DCs) is an important feature in cancer treatment 3. In particular, DCs are crucial in the induction and regulation of innate and adaptive immune responses and have an exceptional capacity to induce cytotoxic T lymphocyte responses, which are essential for tumor control 4. Therefore, exploiting the immunomodulatory function of DCs is currently a crucial strategy to improve immunotherapies against cancer.

The important role of DCs in antigen presentation is aided by several surface receptors that efficiently interact with different ligands and participate in epitope presentation 5. DCs capture antigens in peripheral tissues and then migrate to the secondary lymphoid organs to present the antigens in the context of major histocompatibility complex (MHC) I and MHC II to activate CD8^+^ T cells and CD4^+^ T cells, respectively 6,7. This feature provides immunomodulatory cell-cell contact signals, favoring the release of cytokines that boost immune responses. It is important to take into account that in the context of cancer, there is systemic immunosuppression and a local immunosuppressive microenvironment that impair the effector activities of immune cells 8,9. Facing this obstacle, cancer immunotherapy research may explore different approaches, such as targeting/delivering antigens more specifically to DCs and combining immunotherapies with adjuvants capable of synergistic effects to limit tumor immune escape. Thus, DC targeting is an attractive strategy to increase endogenous antitumor responses and promote cancer eradication. In vivo DC targeting has been shown to be a promising platform capable of improving the immune response 10-12. This approach is based on an antibody-antigen fusion strategy that delivers different target antigens directly to DC subtypes in vivo through different DC receptors, including Fc and C-type lectin receptors 13.

Among numerous surface molecules, the DEC205 receptor is one of the most widely studied for DC targeting strategies. DEC205 (CD205) is a C-type lectin endocytic receptor expressed on thymic epithelial cells and on cDC1s 14,15. cDC1s reside in the T cell areas of the lymphoid organs and have a high capacity to cross-present antigens to CD8^+^ T cells 16. Other approaches to deliver antigens to cDC1s, such as Clec9A, Xcr1 and STxB-based vaccines are known to induce strong antigen-specific B and T cell responses, even in the absence of an adjuvant 16,17. Nonetheless, targeting antigens to DEC205^+^ DCs is usually not sufficient to activate strong antigen-specific immune responses, requiring the use of an additional innate immunity stimulus for cell activation and maturation 18,19.

Immunizations based on anti-DEC205 chimeric mAbs have been effective in generating immune responses against different antigens, such as Her2/neu, HIV gag, the melanoma antigen tyrosinase-related protein (TRP)-2, DENV2 non-structural protein 1 and the *Plasmodium yoelii* circumsporozoite protein 10,20-23. Additionally, selectively targeting DEC205 reduces the amount of antigen required for the generation of T cell immunity and improves antigen presentation almost 100-fold, generating protective T cell immunity 24. Indeed, despite the availability of different DCs-targeting strategies, only DEC-205-targeted vaccines have been evaluated in clinical trials 25.

Human papillomavirus (HPV) is the most common sexually transmitted pathogen worldwide and is associated with nearly all cervical cancer cases and significant numbers of anogenital and head and neck cancers 26. The HPV-16 and HPV-18 strains cause more than 70% of the cases of cervical cancer, which is the fourth most common cancer in women and the fourth most common cause of cancer-related death among women worldwide 27,28. The E7 and E6 oncoproteins are constitutively expressed in HPV-associated tumors and represent clear targets for the development of antigen-specific immunotherapeutic approaches for this type of cancer 29,30. Several therapeutic approaches have been investigated to control tumor growth in both preclinical studies and clinical studies 31-33. However, to date, none of these strategies have yielded strong enough results to justify clinical applications.

In this study, we evaluated a therapeutic immunization strategy against HPV-associated tumors based on an αDEC205 mAb genetically fused to the HPV16-E7 oncoprotein (αDEC205-E7). After characterization of the chimeric antibodies, we evaluated the antitumoral efficacy of the αDEC205-E7 mAb coadministered with poly (I:C) using mice transplanted with TC-1 cells at different anatomical sites. DC targeting by the αDEC205-E7 mAb efficiently induced antitumor cytotoxic T cells (CTLs) and produced strong therapeutic antitumor responses. In addition, targeting the E7 antigen to DEC205^+^ DCs induced long-term immunological memory and prevented tumor relapses.

## Materials and methods

***Construction, expression, and characterization of chimeric antibodies.*** cDNA encoding the E7 sequence was obtained from the plasmid pRE4E7 34 and cloned in-frame with the carboxyl terminus of the heavy chain of a mouse αDEC205 mAb (NLDC145 clone) (kindly provided by Dr Michel C. Nussenzweig, The Rockefeller University) between the 5′ XhoI and 3′ NotI sites. Plasmids encoding the heavy chain and light chain of the mouse αDEC205 mAb were used to transfect human embryonic kidney (HEK) 293T cells (ATCC), and the recombinant mAbs were produced and purified exactly as previously described 10. As a control, the αDEC205 mAb was also produced without any fused antigen. After purification using protein G beads (GE Healthcare), the integrity and specificity of the αDEC205-E7 fusion mAb were determined by SDS-PAGE and Western blotting using anti-mouse IgG-peroxidase (IgG-HRP) (Sigma) and anti-E7 polyclonal antibodies (produced in-house).

***Binding assay*.** CHO cells expressing mouse and human DEC205 receptors (CHOmDEC) (kindly provided by Dr Michel Nussenzweig, The Rockefeller University) were used to perform the binding assay. Each purified antibody (10, 1, or 0.1 (g/mL) was incubated with CHOmDEC cells for 40 min on ice. Next, the cells were washed with FACS buffer (2% fetal bovine serum in PBS) and incubated with an Alexa Fluor 488-conjugated goat anti-mouse IgG mAb (Invitrogen, USA) for 30 min at 4°C. After two washes, the cells were run on an LSRFortessa® flow cytometer (BD Bioscience, USA) and analyzed with FlowJo software.

***Animal studies.*** C57BL/6 mice (female, 6-to-8 weeks old) were purchased from the Facility for SPF (Specific-Pathogen Free) Mouse Production at University of São Paulo Medical School and housed in the Microbiology Department of the University of São Paulo. All the procedures involving animal handling were performed according to protocols approved by the ethics committee for animal experimentation (CEUA 80/2016) and followed the standard rules approved by the National Council for Control of Animal Experimentation (CONCEA).

***Cell lines***. TC-1 cells express the HPV16- E6 and E7 oncoproteins and c-Ha-ras 29. A TC-1-luc cell line was generated by transduction of TC-1 cells with a luciferase-expressing lentiviral vector, as previously described by Kim and collaborators 35. Both cell lines were kindly provided by Dr. T.C. Wu (Johns Hopkins University, USA). Cells were cultured as previously described 32.

***In vivo tumor challenge****.* Subcutaneous (s.c) tumors were established by injection of 10^5^ or 2.5 x 10^5^ TC-1 cells/100 μL/animal into the right mouse flank. Tumor sizes were measured twice per week using a caliper, and survival was followed for at least 60 days. Mice were euthanized when the tumor area reached 200 mm^2^. For an intravaginal tumor model, female C57BL/6 mice were treated with 3 mg of medroxyprogesterone acetate per mouse via s.c injection for diestrus synchronization as previously described 28. Four days later, the mice were intravaginally administered 10^5^ TC-1-luc cells/20 μL/animal. Intravaginal tumor growth was monitored by assessing bioluminescence 5 min after intraperitoneal injection of D-luciferin (Promega, 150 µg/kg of body weight) using the IVIS Imaging System (Caliper, England). Bioluminescence images were analyzed to obtain the total flux values, which refer to the number of photons per second (p/s). To induce an oral tumor model, 5 x 10^4^ TC-1-luc cells/20 μL/animal were injected into the tongue. Tumor growth was monitored by bioluminescence. After 60 days, the mice were reinjected with 10-fold more TC-1 cells. For orthotopic models, mice were euthanized when the number of photons per second (p/s) reached 10^9^-10^10^ or based on the health of the each animal.

***Immunization protocol.*** Mice were immunized with two doses of 10 µg of αDEC205-E7 or αDEC205 mAbs with 50 µg of poly (I:C) via the s.c or i.p. routes on days 3 and 10 after tumor cell grafting. In some experiments, an additional mouse group was immunized with 30 µg of the recombinant E7 protein 31 in the presence, or absence, of 50 µg of poly (I:C). Each dose of treatment was administered on both mice flanks. As controls, animals were left untreated or immunized with 50 μg of poly (I:C) alone.

***In vivo cytotoxicity*.** Splenocytes from naive mice were stained with 0.7 µM and 7.0 µM carboxyfluorescein diacetate succinimidyl ester (CFSE; Invitrogen). The cells stained with 7 µM CFSE were pulsed with 2.5 μg/mL HPV-16 E7 peptide (RAHYNIVTF) for 40 min at 37°C. Equal amounts of the peptide-pulsed (target) and nonpulsed (control) cells were mixed and injected intravenously (2-4 x 10^7^ cells/mouse) 14 days after the last immunization. After 18 h, splenocytes were harvested and analyzed for CFSE staining by flow cytometry on a FACS LSRFortessa (BD Biosciences, USA).

***Flow cytometry analysis****.* The blood, spleen and tumor-draining lymph nodes were collected 7 or 14 days after the last immunization. Cells were incubated at a concentration of 1.5 x 10^6^ cells per well and stimulated overnight at 37°C in the presence of 10 µg/mL brefeldin A (GolgiPlug; BD Biosciences, USA) and 2 μg/mL anti-CD28 antibody (BD Biosciences, USA) with 1.5 μg/mL E7-specific RAHYNIVTF peptide (amino acids 49-57) at a final concentration of 300 ng/well. After the incubation, cells were stained with the Live/Dead Fixable Aqua vitality dye, an APC-Cy7-conjugated anti-CD3 antibody, and a PE-Cy7-conjugated anti-CD8 antibody (BD Biosciences, USA). For memory phenotyping assays, blood cells were also stained with PE-conjugated anti-CD44 and FITC-conjugated anti-CD62L antibodies (BD Biosciences, USA) for 30 min at 4°C. Cells were fixed, permeabilized (Cytofix/Cytoperm) and stained with a BV421-conjugated anti-IFN-γ antibody for 30 minutes at 4°C. All samples were run on an LSRFortessa® flow cytometer (BD Bioscience, USA) and analyzed with FlowJo software.

***Data analysis*.** A t-test or ANOVA followed by the Bonferroni posttest were performed when groups were compared. Log-rank tests were performed whenever survival curves were compared. All p values < 0.05 were considered statistically significant.

## Results

### The chimeric αDEC205-E7 mAb retains its ability to bind to the DEC205 receptor

**Figure 1:**
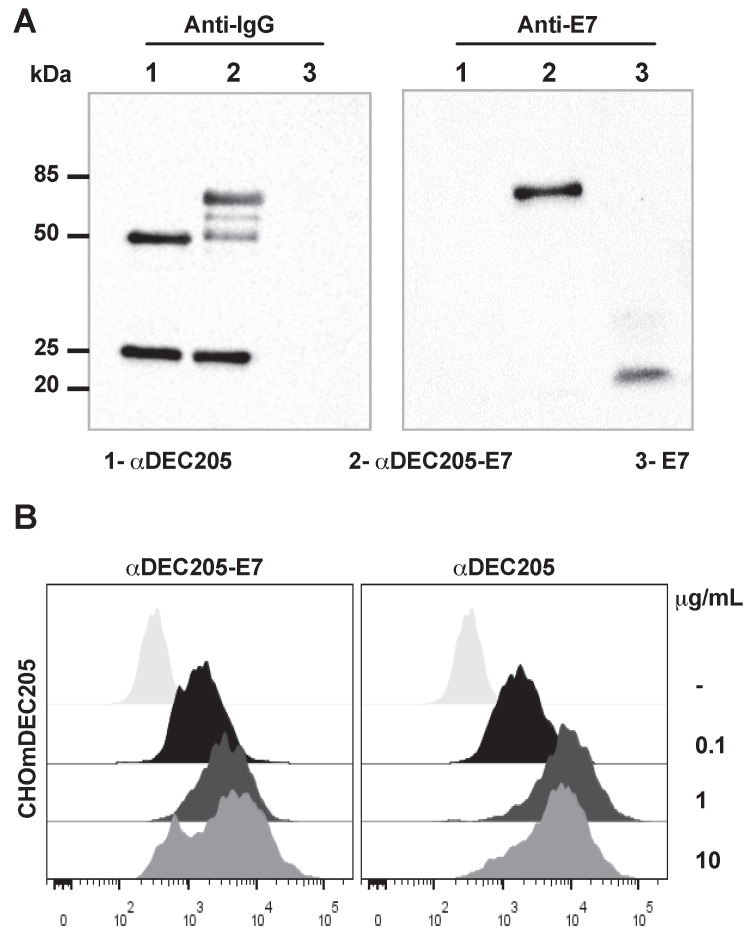
** The αDEC205-E7 mAb binds to the murine DEC205 receptor.** (A) The mouse αDEC205 mAb (line 1), αDEC205-E7 mAb (line 2) and recombinant E7 protein (line 3) were separated on a 12% polyacrylamide gel (shown in Figure S1) and evaluated by immunoblotting with a peroxidase-labeled goat anti-mouse IgG antibody (gel on the left) and an anti-E7 mouse polyclonal antibody (gel on the right). (B) CHO cells expressing the mouse DEC205 receptor were incubated with 0.1, 1, or 10 µg/ml αDEC205-E7 mAb (dot plot on the left) or αDEC205 mAb (dot plot on the right). Binding was detected by flow cytometry using an Alexa Fluor 488-conjugated anti-mouse IgG mAb.

To build a therapeutic vaccine capable of specifically targeting the HPV-16 E7 oncoprotein to a subset of DCs, we cloned an E7-encoding sequence in-frame with a sequence encoding the murine αDEC205 heavy chain. As a control, we also produced the αDEC205 mAb without any fused antigen. The construction of the αDEC205-E7 mAb produced a chimeric heavy chain fused with the E7 sequence with an expected molecular weight of approximately 75 kDa (Figure S1). An E7-specific polyclonal antibody recognized the αDEC205-E7 heavy chain but not that of unfused αDEC205, which can be seen in a Western blot probed with an anti-IgG antibody (Figure 1A).

To confirm the specificity of the binding between the αDEC205-E7 mAb and DEC205, we incubated the αDEC205-E7 mAb to CHO cells expressing the DEC205 mouse receptor (CHOmDEC205) and labeled the cells with an Alexa Fluor 488-conjugated goat anti-mouse IgG mAb. As shown in Figure 1B, the αDEC205-E7 mAb specifically bound CHO cells expressing the murine DEC205 receptor (Figure 1B), even at low concentrations. These results demonstrate that the αDEC205-E7 mAb retained the capacity to bind specifically to the murine DEC205 receptor.

### Immunization of tumor-bearing mice with the αDEC205-E7 mAb induced a robust antitumor response with generation of IFN-γ-producing cytotoxic CD8^+^ T cells

**Figure 2:**
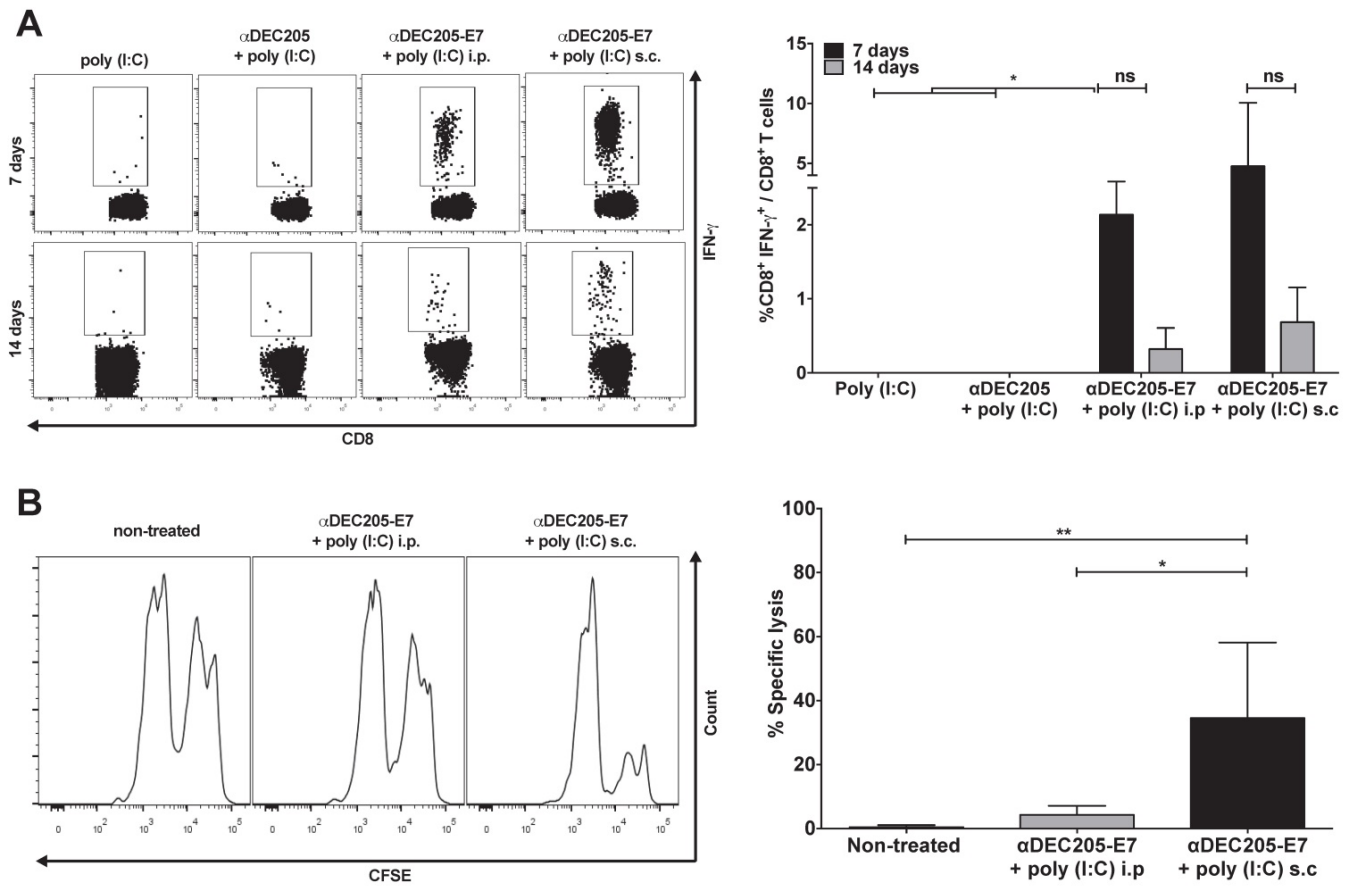
** Induction of E7-specific CD8^+^ T cell IFN-γ^+^ production and in vivo cytotoxic effects elicited in C57BL/6 mice immunized with the αDEC205-E7 mAb via the s.c. or i.p. route.** C57BL/6 mice were engrafted with 2.5 x 10^5^ TC-1 cells (day 0) and immunized on days 3 and 10 with 10 µg of αDEC205-E7 mAb admixed with 50 µg of poly (I:C) via either the i.p. or s.c route, as indicated. Mice were also immunized with two doses of the αDEC205 mAb admixed with poly (I:C) or only poly (I:C) for comparison. Blood samples were collected 7 and 14 days after the last immunization, and PBMCs were stimulated overnight with a peptide corresponding to the HPV-16 E7 K^b^ MHC class I-restricted immunodominant epitope. (A) Dot plots on the left side of the figure are a representative for gated CD8^+^ IFN-γ^+^ cells. The graph on the right side shows the percentage of CD8^+^ IFN-γ^+^ cells determined after subtracting the values obtained with unstimulated cells. (B) Fourteen days after the last immunization, mice were injected with CFSE-labeled splenocytes pulsed with or without the E7-derived peptide. On the left side of the panel, representative histograms of the in vivo cytotoxicity assay are shown. The graph to the right shows the in vivo cytotoxic activity represented by the percentage of target-cell lysis (n=5). Experiments were repeated twice. Statistical significance: *p < 0.05, **p < 0.01, (ns) non-significant difference.

After validating the αDEC205-E7 mAb in vitro, our next step was to test two in vivo immunization routes to assess the best strategy for induction of HPV-16 E7-specific CD8^+^ T cell responses. Mice were initially grafted with 2.5 x 10^5^ TC-1 cells s.c and subsequently immunized with two doses of the αDEC205-E7 mAb coadministered with poly (I:C) either via the intraperitoneal (i.p) or s.c route (Figure 2). As a control, mice were immunized with two i.p. doses of the αDEC205 mAb or poly (I:C) adjuvant only. Mice immunized with αDEC205-E7 mAb + poly (I:C), via either the i.p or s.c route, exhibited increased numbers of IFN-γ-producing CD8^+^ T cells when probed on days 7 and 14 after the last immunization (Figure 2A). Furthermore, mice immunized by the s.c route exhibited stronger cytotoxic responses than the i.p-immunized mice (Figure 2B). In addition, the mice s.c immunized with the αDEC205-E7 mAb showed enhanced tumor growth control compared with the i.p-immunized mice (Figure S2A-C). Thus, the s.c administration route was chosen for subsequent experiments.

### Targeting E7 to DCs via the DEC205 receptor induces antitumor protection in mice s.c transplanted with tumor cells expressing HPV-16 oncoproteins

**Figure 3:**
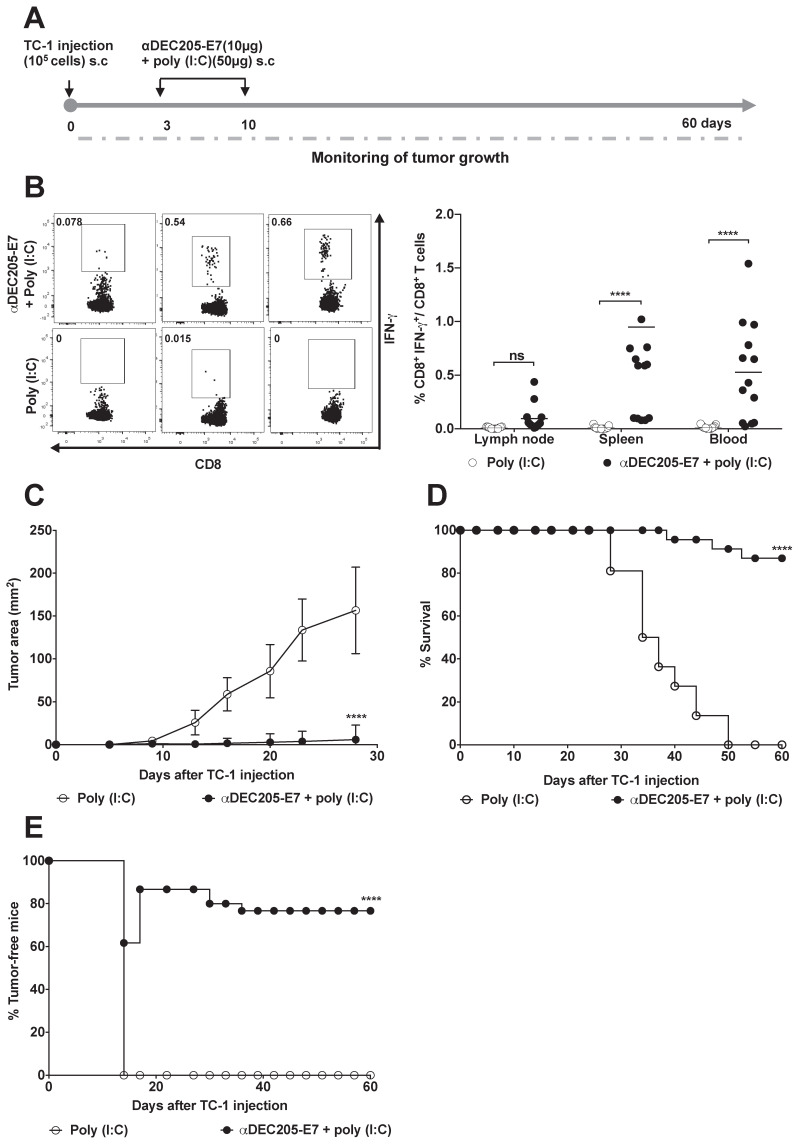
** Immunization with the αDEC205-E7 mAb induces E7-specific CD8^+^ T cell responses and confers efficient therapeutic antitumor effects.** C57BL/6 mice were engrafted in the right flank with 10^5^ TC-1 cells (day 0) and s.c. immunized at days 3 and 10 with 10 µg of αDEC205-E7 mAb admixed with poly (I:C). An additional mouse group was immunized with only poly (I:C). Splenocytes, PBMCs, and tumor-draining lymph nodes cells were collected 7 days after the last immunization and stimulated with a synthetic peptide corresponding to the MHC class I-restricted E7-specific epitope overnight. The cells were subsequently labeled with fluorophore-conjugated anti-CD3, anti-CD8, and anti-IFN-γ mAbs for flow cytometry analysis. Tumor growth was monitored 2-3 times per week for 60 days. (A) Experimental design. (B) Representative dot plots (left) and percentages (right) of CD8^+^ IFN-γ^+^ T cells determined after subtracting the values obtained with unstimulated cells. (C) tumor size over time (two-way ANOVA). (D) Survival percentage (log-rank-Mantel-Cox). (E) Percentage of tumor-free mice over time. Data are expressed as the mean ± SD from three experiments with similar results (n= 21-23 mice/group). Statistical significance:***p < 0.001, ****p < 0.0001, (ns) non-significant.

Next, we investigated whether the DEC205-based DC-targeting approach would induce therapeutic antitumor effects on TC-1 cells s.c transplanted into C57BL/6 mice. Mice were immunized with two doses of 10 µg of αDEC205-E7 mAb + poly (I:C) on days 3 and 10 after tumor cell transplantation. When the immunization protocol starts 3 days after tumor cell engraftment, the tumor mass cannot be detected yet. We can observe the palpable tumor around 10 days post-TC-1 cells inoculation (Figure 3). As control groups, mice were inoculated only with the poly (I:C) adjuvant (Figure 3A), E7 or E7 plus poly (I:C) (Figure S3A-B). One week after the last immunization, splenocytes, blood and tumor-draining lymph nodes were collected, and cells were cultured in the presence of a synthetic peptide corresponding to the immunodominant MHC class I-restricted E7 epitope. Analysis of intracellular IFN-γ production showed that immunization with the αDEC205-E7 mAb + poly (I:C) induced a peak IFN-γ response 7 days after immunization (Figure 3B). Activation of IFN-γ-producing CD8^+^ T cells was detected in the splenocytes, blood and tumor-draining lymph nodes of the mice immunized with the αDEC205-E7 mAb, with the highest CD8^+^ T cell responses observed in the blood. The secretion of IFN-γ by splenocytes upon stimulation with the immunodominant peptide was also observed by Cytometric Bead Array (CBA) in the αDEC205-E7 immunized group (Figure S4). Corroborating the cellular activation data, the tumor sizes (tumor-bearing mice) of mice immunized with the αDEC205-E7 mAb + poly (I:C) were significantly smaller than those of mice immunized with poly (I:C) (Figure 3C). In addition, 80% (18 of 23) of the mice immunized with the αDEC205-E7 mAb + poly (I:C) survived until the end of the follow-up period (Figure 3D). Notably, administration of the αDEC205-E7 mAb + poly (I:C) conferred approximately 80% (18 of 23) tumor protection (tumor-free animals). Importantly, even when the treatment started with tumors still undetectable, immunization with poly (I:C) alone, recombinant E7, or E7 + poly (I:C) did not control the tumor mass as efficiently as did the administration of poly (I:C) combined with anti-DEC205-E7 (Figure 3E and Figure S3A). As the tumor microenvironment is already installed, if the immunotherapy is not properly efficient, the tumor is likely to grow. Taken together, these results indicate that s.c immunization with the αDEC205-E7 mAb + poly (I:C) activates systemic E7-specific CD8^+^ T cell responses and efficiently confers therapeutic antitumor effects.

### Immunization with the αDEC205-E7 mAb confers therapeutic antitumor protection in two orthotopic tumor models

**Figure 4:**
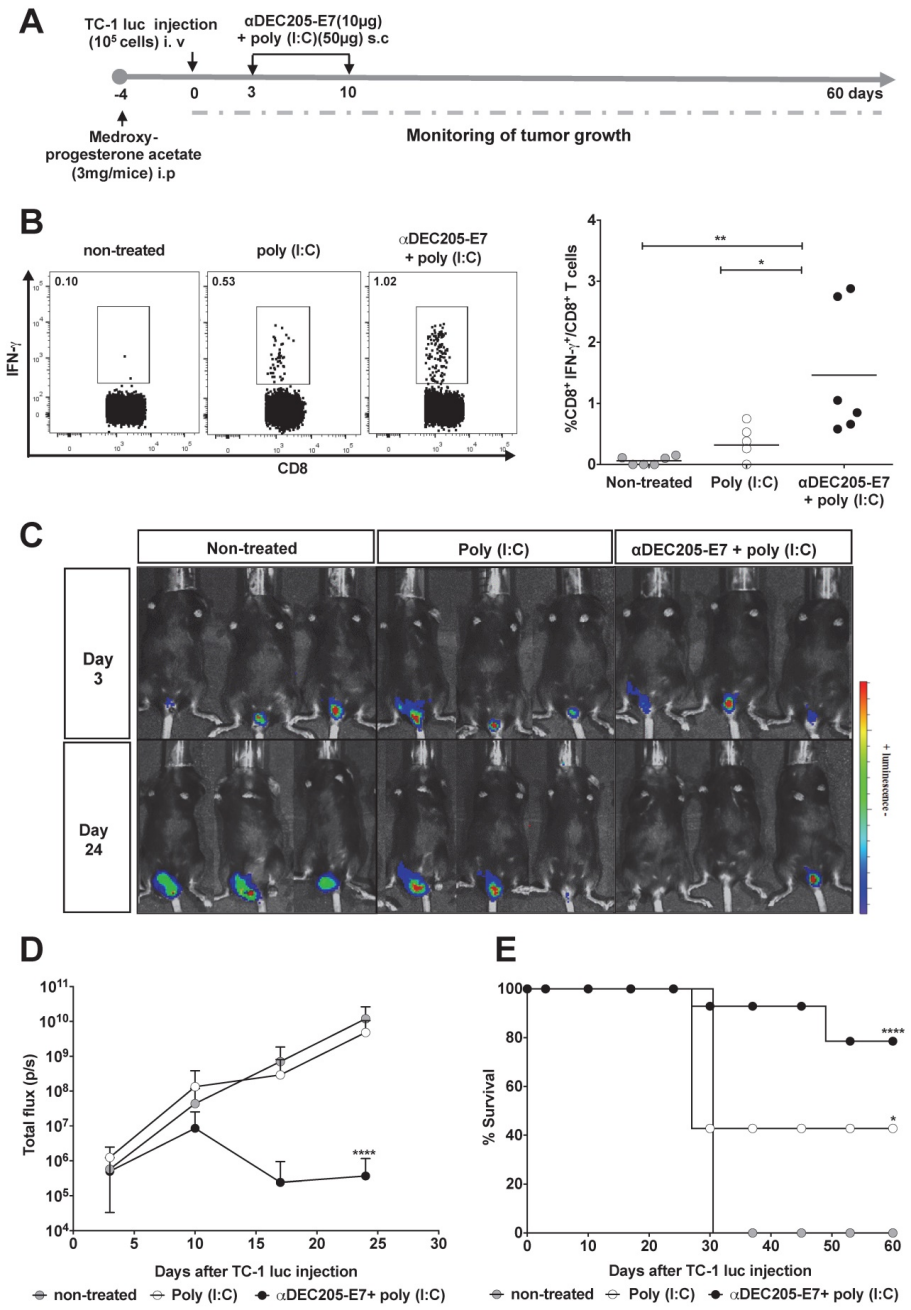
** Immunization with the αDEC205-E7 mAb induces E7-specific CD8^+^ T cell responses and therapeutic antitumor effects in mice transplanted with TC-1 cells at different mucosal sites.** Female C57BL/6 mice received medroxyprogesterone acetate (3 mg/mouse) and, 4 days later, were engrafted with 10^5^ TC-1-luc cells in the genital mucosa. Three and 10 days later, the animals were s.c immunized with 10 µg of αDEC205-E7 mAb admixed with poly (I:C). Intravaginal tumor growth was monitored once a week. Blood samples were collected 7 days after the last immunization, and cells were stimulated overnight with a peptide corresponding to the immunodominant K^b^ MHC class I-restricted HPV-16 E7-specific epitope. (A) Experimental design. (B) Representative dot plots (left) and the percentage of IFN-γ-secreting CD8^+^ T cells (right), which was determined after subtracting the values recorded for unstimulated cells. (C) Representative images of the luciferase activity in mice measured 5 min after luciferin injection. (D) Quantification of the total photon flux (p/s) emitted during the luminescence reaction (two-way ANOVA). (E) Survival percentage (log-rank-Mantel-Cox) (n = 14). Experiments were reproduced two times. Statistical significance: *p < 0.05, ** p < 0.01, **p < 0.001, ns = nonsignificant.

**Figure 5:**
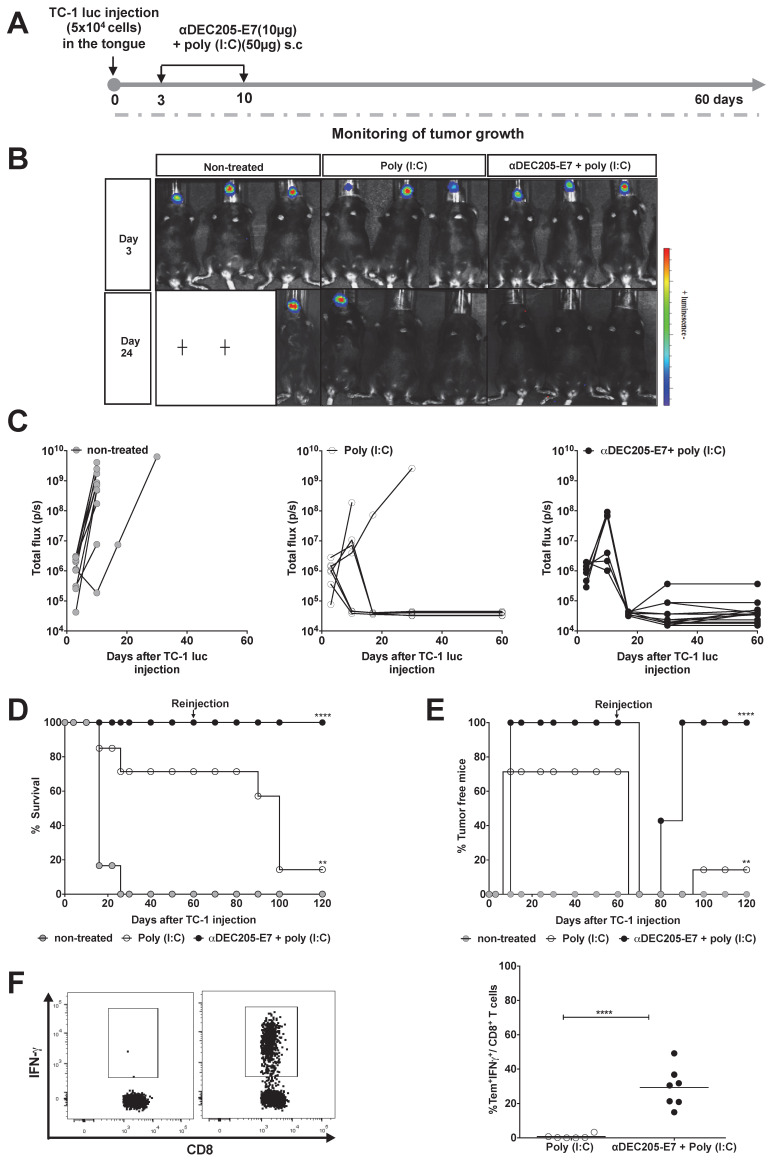
** Immunization with the αDEC205-E7 mAb prevents tumor relapse and induces robust activation of E7-specific effector memory CD8^+^ T cells.** C57BL/6 mice were engrafted with 5x10^4^ TC-1-luc cells in the tongue and s.c immunized 3 and 10 days later with 10 µg of αDEC205-E7 mAb admixed with poly (I:C) or treated with only poly (I:C). Tumor rechallenge was performed 60 days after the first cell implantation using 10-fold more TC-1-luc cells. Blood cells were collected 7 days after rechallenge and stimulated in vitro overnight with the E7_39-47_ peptide. The gating strategy is shown in S4. (A) Experimental design. (B) Representative luciferase activity 5 min after luciferin injection. (C) Quantification of the total photon flux (p/s). (D) Survival percentage. (E) Percentage of tumor-free mice over time (log-rank-Mantel-Cox) (n=14). (F) Representative dot plots (left) and the percentage of IFNγ-producing effector memory CD8^+^ T cells (CD8^+^CD44^+^CD62L^-^) (right) (Student's t-test) (n = 5-7). Experiments were reproduced two times. Statistical significance: **p < 0.01, ****p < 0.0001.

We next evaluated whether immunotherapy confers antitumor protection using two orthotopic tumor models (the vaginal epithelium and tongue) that more closely resemble the usual clinical conditions. For the intravaginal and tongue models, mice were engrafted with luciferase-expressing TC-1 cells and treated with the same vaccination regimen (Figures 4A and 5A, respectively). We measured luciferase activity 3 days after tumor cell engraftment and followed tumor growth for 60 days. In accordance with the previous results, immunization with the αDEC205-E7 mAb + poly (I:C) induced activation of E7-specific IFN-γ-producing CD8^+^ T cell responses in the blood of mice transplanted intravaginally with TC-1-luc cells (Figure 4B). However, in contrast to the results generated in mice s.c. transplanted with TC-1 cells, mice treated with only poly (I:C) showed a slightly increased number of IFN-γ-producing CD8^+^ T cells (Figure 4B). Regarding intravaginal tumor development, mice immunized with the αDEC205-E7 mAb + poly (I:C) controlled intravaginal tumor growth more efficiently than the mice treated with only poly (I:C) or left untreated (Figures 4C-D and S5). Mice immunized with αDEC205-E7 mAb + poly (I:C) showed approximately 80% survival at the end of the observation period. In contrast, only 40% of the mice treated with poly (I:C), and no mice left untreated, survived until the end of the observation period (Figure 4E).

Similar experiments were carried out with mice engrafted with TC-1-luc cells in the tongue (Figure 5). Following the same immunization protocols and follow-up periods (Figure 5A), mice immunized with the αDEC205-E7 mAb + poly (I:C) showed complete tumor protection. Unexpectedly, 75% of the mice treated with poly (I:C) alone exhibited tumor growth control (Figures 5B-C and S6). These results highlight the specific features of the immune responses associated with this mucosal site.

### Immunization with the αDEC205-E7 mAb prevents tumor recurrence and promotes activation of an effector memory CD8^+^ T cell response

Since we did not observe a significant difference between the antitumor protection of αDEC205-E7 mAb + poly (I:C) and poly (I:C) alone in the tongue tumor model, we decided to investigate whether the combined immunotherapy would induce long-lasting immune protection. To assess long-lasting protection, we investigated the impact of αDEC205-E7 mAb + poly (I:C) immunization on the incidence of tumor relapse in the tongue tumor model. For these experiments, mice that were tumor-free after immunization with the αDEC205-E7 mAb + poly (I:C) or poly (I:C) alone were rechallenged with TC-1 luc cells at a dose 10-fold higher than that used for the initial tumor challenge. TC-1 cell grafts were inoculated in the tongue 60 days after the first tumor challenge. All mice immunized with the αDEC205-E7 mAb remained protected following rechallenge (Figure 5D) and survived for an additional period of 60 days (total observation period of 120 days) (Figure 5E). Under this experimental condition, although capable of controlling the primary tumors, the mice treated with poly (I:C) showed only 14% protection against tumor growth at the end of the observation period (Figure 5D-E).

Finally, to determine whether memory T cells are induced after immunization, we determined if memory effector CD8^+^ T cells were present by incubating blood cells with the E7_39-47_ peptide one week after reinjection of TC-1-luc cells into the tongue. The results demonstrated that mice immunized with the αDEC205-E7 mAb + poly (I:C) efficiently mounted an IFN-γ-producing effector memory CD8^+^ T cell response (CD8^+^CD44^high^CD62L^-^, Figure S7), while animals treated with poly (I:C) alone did not (Figure 5F). These results indicate that immunization with the αDEC205-E7 mAb + poly (I:C) induced antitumor immunity and conferred immunological memory capable of controlling tumor relapses.

## Discussion

In the current study, we investigated the therapeutic antitumor effects conferred by targeting the HPV16 E7 oncoprotein to DCs with the αDEC205 mAb. The antitumor effects were measured using TC-1 cells, which were used to generate solid tumors at different anatomical sites. In addition to s.c. implantation, injection at two different mucosal sites, the tongue and vagina, was performed with TC-1 cells expressing luciferase (TC-1 luc cells). Our results demonstrated that targeting the HPV-16 E7 oncoprotein to DCs with the αDEC205 mAb generated efficient activation of an E7-specific CD8^+^ T cell response and promoted enhanced therapeutic antitumor effects on TC cells in all three tumor models. More importantly, immunotherapy induced long-term immunological memory and prevented tumor relapse in rechallenged mice.

E7 targeting to DCs through the DEC205 receptor has been explored previously 36. Z. Liu et al. showed the efficacy of delivering E7 to the DEC205 receptor with a DEC205-specific single-chain variable fragment in a prophylactic setting, protecting mice s.c. challenged with TC-1 cells. However, an efficient DEC205-targeting HPV E7 vaccine based on a full chimeric antibody has not been reported. In addition, the present study is the first to demonstrate the impact of this approach on tumors at three different anatomical sites using both non-orthotopic and orthotopic tumor models. In a therapeutic setting, administration of the αDEC205-E7 mAb plus poly (I:C) protected mice from tumor growth at all sites analyzed.

The E7 oncoprotein represents the most appropriate antigen for the development of HPV immunotherapies. However, immunization with this antigen alone exhibits low immunogenicity and is unable to induce an efficient T cell response 31,37. Additionally, in the present and previous studies, we reported that immunization with the E7 protein alone or co-administered with adjuvants is not capable of controlling tumor growth in TC-1 cell-transplanted mice 31. In this study, our data showed that E7 fused to the αDEC205 mAb was capable of reversing immune tolerance to E7 and activating cytotoxic T lymphocytes, generating high frequencies of systemic E7-specific CD8^+^ T cells. Furthermore, immunization with the αDEC205-E7 mAb elicited complete or almost complete therapeutic antitumor protection in mice engrafted with TC-1 tumor cells at different anatomical sites. Our findings indicate that the DEC205-targeting vaccination platform can efficiently overcome the low endogenous immunogenicity of the E7 protein and induce strong antitumor immunity. Moreover, targeting antigens to DCs by means of the DEC205 receptor proved to be a more potent therapeutic approach than conventional non-DC targeting protein-based vaccines.

Several studies have shown that targeting antigens to DEC205^+^ DCs without providing a maturation stimulus is insufficient to stimulate an immune response; additional combination with immunostimulatory signals or adjuvants is required to promote antitumor immune responses 38,39. In this regard, the immunization protocols used in this study included the use of poly (I:C) as an adjuvant, as poly (I:C) has been shown to be the most potent immune activator of DCs in the context of αDEC205 mAb administration 22,23,40. The administration of poly (I:C) alone was capable of controlling approximately 50% and 75% of tumors in the intravaginal and oral tumor models, respectively. We reasoned that these results may be explained by the reported antitumor activity of poly (I:C), which binds to TLR3 and leads to the activation of NF-kB and production of type I interferons by different cells 41. Furthermore, poly (I:C) can mediate tumor cell apoptosis and activate NK cells 42,43 Currently, TLR3 ligands are being tested either alone or in combination with chemotherapeutics or immunotherapeutics in several cancer immunotherapy trials. However, these ligands are not yet approved for clinical application. Based on their abilities to activate the immune system, other TLR agonists have been tested for the treatment of cancer, such as the TLR7 ligand imiquimod, which is efficient in treating HPV-induced neoplasias 44,45. Thus, in vivo targeting of DCs with TLR agonists has produced promising results in both preclinical studies and clinical trials.

In the present study we observed that, despite the lack of effects on s.c. transplanted tumor cells, poly (I:C) alone was capable of inducing partial antitumor effects in mice challenged with TC-1-luc cells at orthotopic mucosal sites. Nonetheless, administration of poly (I:C) does not generate long lived protective immunity neither in these tumor models nor confers immunological memory responses regarding the anti-tumor effects. One point to consider is the observed anti-tumor effects of poly (I:C) in orthotopic tumor models based on TC-1-luc cells. This cell lineage has been previously reported to be more easily treated than conventional TC-1 cell strain after treatment of mice with a peptide-based vaccine in combination with poly (I:C) 46. However, we believe that further studies aimed to evaluate the synergic effects of different poly (I:C) derivatives in combination with anti-tumor vaccines may bring relevant information regarding translation into clinical conditions.

Previous reports have shown that the efficacy of therapeutic anti-tumor vaccines may differ depending on the immunization route and tumor types 47. In the case of epithelial tumors, such as those induced by HPV, induction of mucosal immunity is usually quite relevant for development of an effective anti-tumor immunity 48,49. For example, intranasal (i.n.) vaccination has been particularly efficient to induce mucosal immune responses and protection to head and neck or lung cancers 48. Nonetheless, other studies demonstrate that parenteral immunization may lead to more efficient anti-tumor immunity 50. In our study, administration of αDEC205-E7 mAb by the s.c route was efficient to induce immune responses capable to eradicate both mucosal (genital and lingual) and subcutaneous tumors. These results corroborate recent studies demonstrating that the s.c. administration of a bivalent therapeutic vaccine induced full tumor regression in mice bearing orthotopic genital HPV tumors 46. Indeed, attempts to deliver αDEC205-E7 mAb via the i.n. route failed to induce significant antitumor protection (unpublished observation). These results suggest that mucosal delivery of recombinant DC-targeting mAbs will require additional adaptations in order to cross the epidermal barrier and promote efficient immune responses against HPV-associated tumors.

The αDEC205 mAb is a molecular vector for efficient delivery of antigens to a specific subset of DCs that express the DEC205 receptor. Several other mAbs are presently being used as immunotherapies to treat cancer and other illnesses 51. Nonetheless, in contrast to immune checkpoint inhibitor mAbs, the αDEC205-E7 mAb showed no side effects. Although there is no doubt about the effectiveness of immune checkpoint inhibitors as a cancer therapy, valid concerns about the risks of immune-mediated toxic effects and acquired resistance as well as the high cost of this type of therapy represent real challenges limiting the widespread use of this technology 51. Another important fact to be highlighted is the inherent feature of passive immunotherapy, which includes the use of immune checkpoint mAb-based therapies and even T cell-based therapies; in both cases, long-lasting immune memory cells are not activated 51-53. This feature could partially explain the failure of these therapies in preventing tumor relapses in patients treated with anti-CTLA-4 and/or anti-PD-1/L1 mAbs or CAR T cells. In contrast, anticancer vaccines, such as those based on the αDEC205-E7 mAb, provide new insight into tumor therapy.

Active immunotherapies are capable of stimulating antitumor T cells and generating long-term immunity. In this study, immunization with the αDEC205-E7 mAb induced strong activation of E7-specific CD8^+^ T memory cells and prevented tumor relapse in mice rechallenged with TC-1 cells in the tongue. Our data demonstrate the high efficiency of this approach in controlling primary tumors and preventing tumor relapse via active stimulation of tumor-specific immune responses, including immunological memory. Regarding the clinical use of αDEC205 mAbs, a trial based on an anti-human DEC205 antibody fused to NY-ESO-1 reported that the vaccine was well-tolerated and induced cellular immune responses against NY-ESO-1-expressing tumors 25. Thus, the immunization approach based on the concept of targeting antigens to DCs may be applicable for different kinds of tumors.

Collectively, our observations illustrate the potential of antigen targeting to DCs as a platform for the development of therapeutic antitumor vaccines. Our data provide preclinical evidence that delivery of the E7 oncoprotein to DEC205^+^ DCs represents a promising strategy for the control of HPV-associated tumors. Ongoing research expanding this approach holds promise for continued substantive contributions to the field of cancer immunotherapy.

## Supplementary Material

Supplementary materials and methods, figures.

## Abbreviations

ATCC: american type culture collection
CAPES: coordenação de aperfeiçoamento de pessoal de nível superior
CEUA: ethics committee on the use of animals in experimentation
CONCEA: national council for control of animal experimentation
c-Ha-ras: harvey rat sarcoma viral oncogene homolog
CNPq: Conselho Nacional de Desenvolvimento Científico e Tecnológico
E6: early protein 6
E7: early protein
FAPESP: fundação de amparo à pesquisa do estado de São Paulo
USP: university of São Paulo
