# Supplementary material for: Antigen Delivery to DEC205+ Dendritic Cells Induces Immunological Memory and Protective Therapeutic Effects against HPV-Associated Tumors at Different Anatomical Sites
Source: Int J Biol Sci. 2021 Jul 13;17(11):2944–56. doi: 10.7150/ijbs.57038 (PMC8326119; doi:10.7150/ijbs.57038)
Supplement: Supplementary file 1 — Supplementary materials and methods, figures. [file ijbsv17p2944s1.pdf]

SUPPLEMENTARY MATERIAL

Materials and methods

Cytokine Beads Array (CBA). Spleen were collected 7 or 14 days after the last immunization. Cells were incubated at a concentration of 1.5 x 10^6 cells per well and stimulated overnight at 37°C in the presence of 2 μg/mL anti-CD28 antibody (BD Biosciences, USA) and 1.5 μg/mL E7-specific RAHYNIVTF peptide (amino acids 49–57) at a final concentration of 300 ng/well. After 12 hour of incubation, the levels of IFNγ in cell culture supernatants were analyzed using a cytometric bead array kit (CBA) (Mouse Th1/Th2/Th17 Cytokine Kit, Becton Dickinson, San Jose, CA).The samples were run on an LSRFortessa® flow cytometer (BD Bioscience, USA) acquired and analyzed using FCAP array software (BD Biosciences).

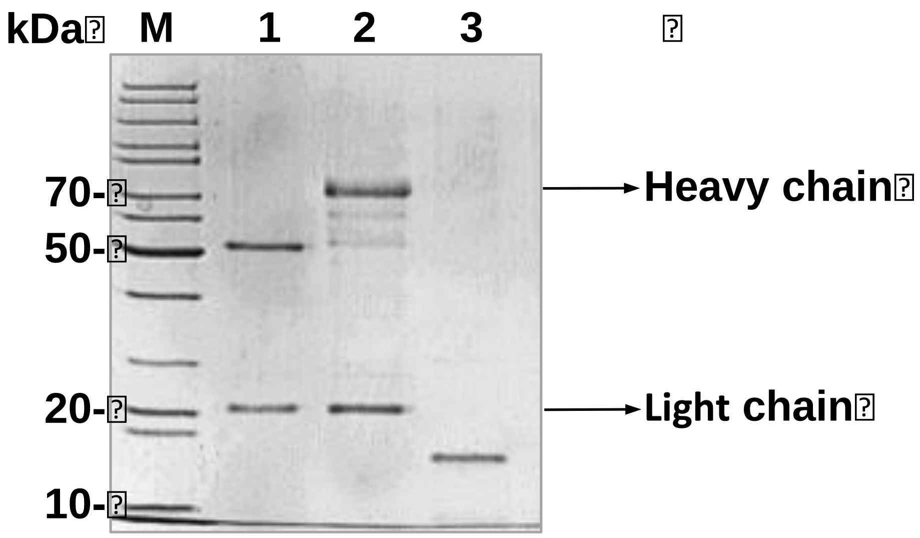

Figure S1. SDS-PAGE gel electrophoresis of αDEC205-E7 mAbs. The mouse anti-DEC205 mAb, anti-DEC205 fused to E7 protein and E7 protein were run on 12% SDS-PAGE under reducing conditions and stained with Coomassie Blue. The heavy (HC) and light (LC) chains of the following antibodies are shown as indicated in the figure. Line 1: αDEC205, line 2: αDEC205-E7, line 3: E7. M: molecular weight marker.

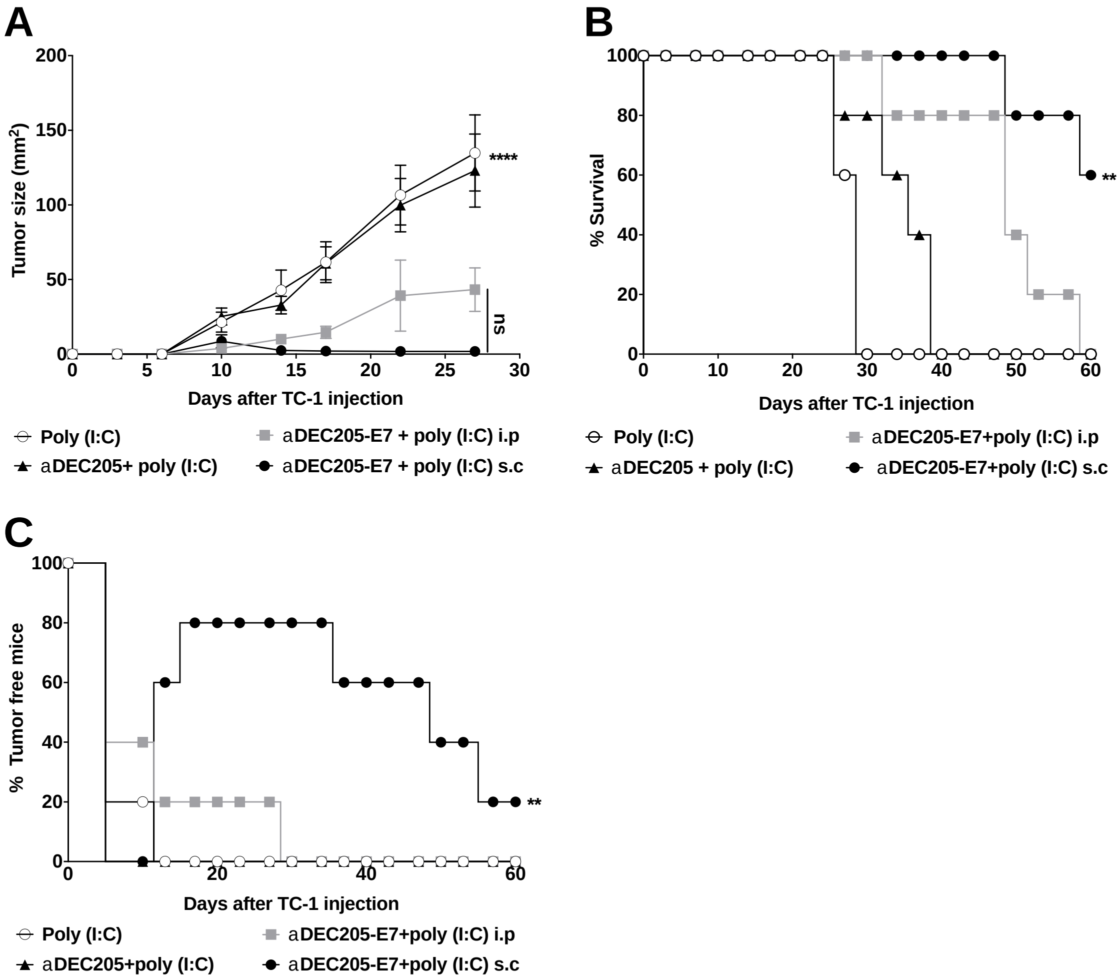

Figure S2. Subcutaneous administration of αDEC205-E7 promotes better antitumor effects than intraperitoneal route in TC-1 model. C57BL/6 mice were grafted with 2.4 x 10^5 TC-1 cells subcutaneously on the right flank and immunized 3 and 10 days later with 10 µg αDEC205-E7 mAbs in the presence of 50 µg poly (I:C) by intraperitoneal (i.p) or subcutaneous (s.c) routes. As control group, mice were immunized with two doses of the αDEC205 plus poly (I:C) or poly (I:C) alone intraperitoneally. Tumor growth was monitored 2–3 times per week for a period of 60 days (A) Estimated tumor size over time (B) Percentage of survival or (C) Percentage of tumor-free mice over time (log-rank–Mantel–Cox). Experiments were reproduced two times. (n=5) $^{**}p < 0.01$, $^{****}p < 0.0001$, ns =Non-significant.

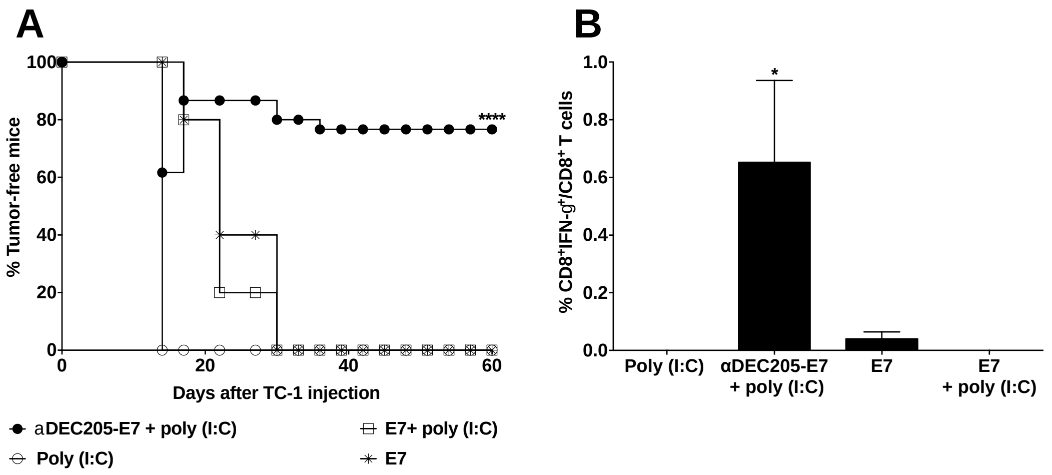

Figure S3. Antitumor effects of αDEC205-E7 in mice s.c. transplanted with TC-1 cells. (A) Anti-tumor therapeutic effects measured in mice transplanted with TC-1 cells. For C57BL/6 mice immunized with E7 or E7 plus poly (I:C) the animals were challenged with 7.5×10^4 TC-1 cells, and subsequently treated with two s.c doses (days 1 and 8 after challenge) containing 30 µg of E7 and 50 µg of poly (I:C) (n=5). For mice immunized with αDEC205-E7, animals were challenged with 10^5 TC-1 cells, and subsequently treated with two s.c doses (days 3 and 10 after challenge) containing 10 µg of αDEC205-E7 mAb admixed with 50 µg of poly (I:C) (n=7, log-rank–Mantel–Cox). (B) Induction of IFN-γ-producing E7-specific $CD8^+$ T cell responses in mice treated with αDEC205-E7. The same mouse groups described in (a) were bled 7 days after the last vaccine dose and analyzed by flow cytometry as reported in M&M. Experiments were reproduced three times. Statistical significance was determined by one-way ANOVA. *$p < 0.05$, ****$p < 0.0001$.

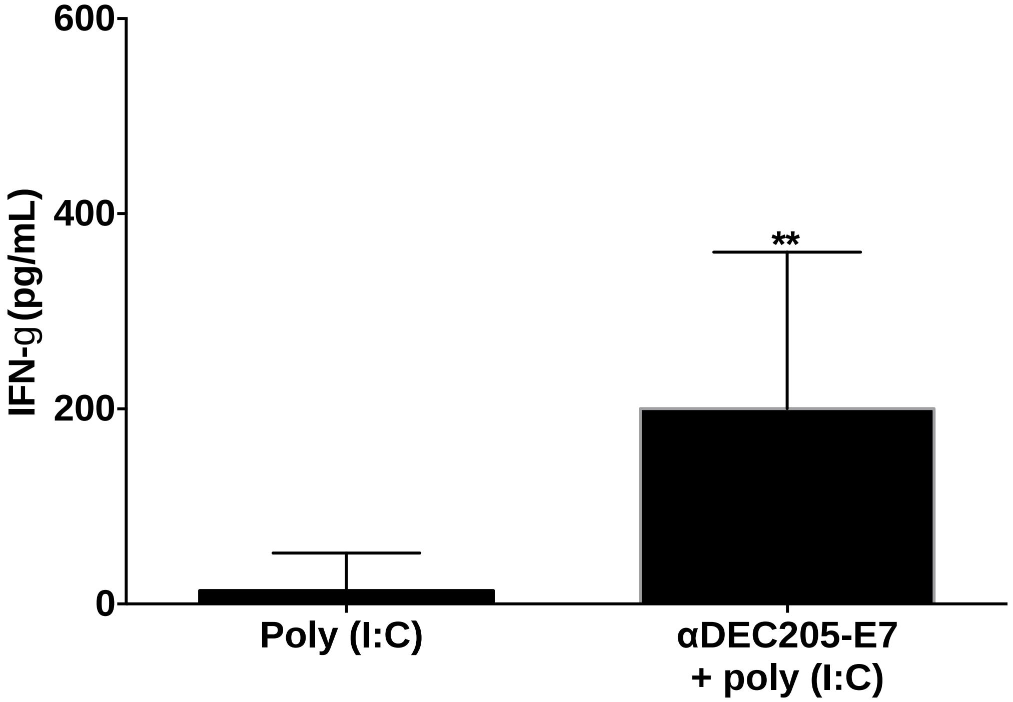

Figure S4. Detection of IFNγ-secreted by splenocytes stimulated with an MHC class I-restricted E7-specific epitope. C57BL/6 mice were engrafted in the right flank with 10^5 TC-1 cells (day 0) and s.c. immunized at days 3 and 10 with 10 µg of αDEC205-E7 mAb admixed with poly (I:C). An additional mouse group was immunized with only poly (I:C). Splenocytes were collected 7 days after the last immunization and stimulated with a synthetic peptide corresponding to the E7 peptide overnight. IFNγ-secreted was determined by Cytometric Bead Array (CBA). Analyses were performed using FCAP array software. (n=9). Statistical significance was determined by t-test. $^{**}p < 0.01$.

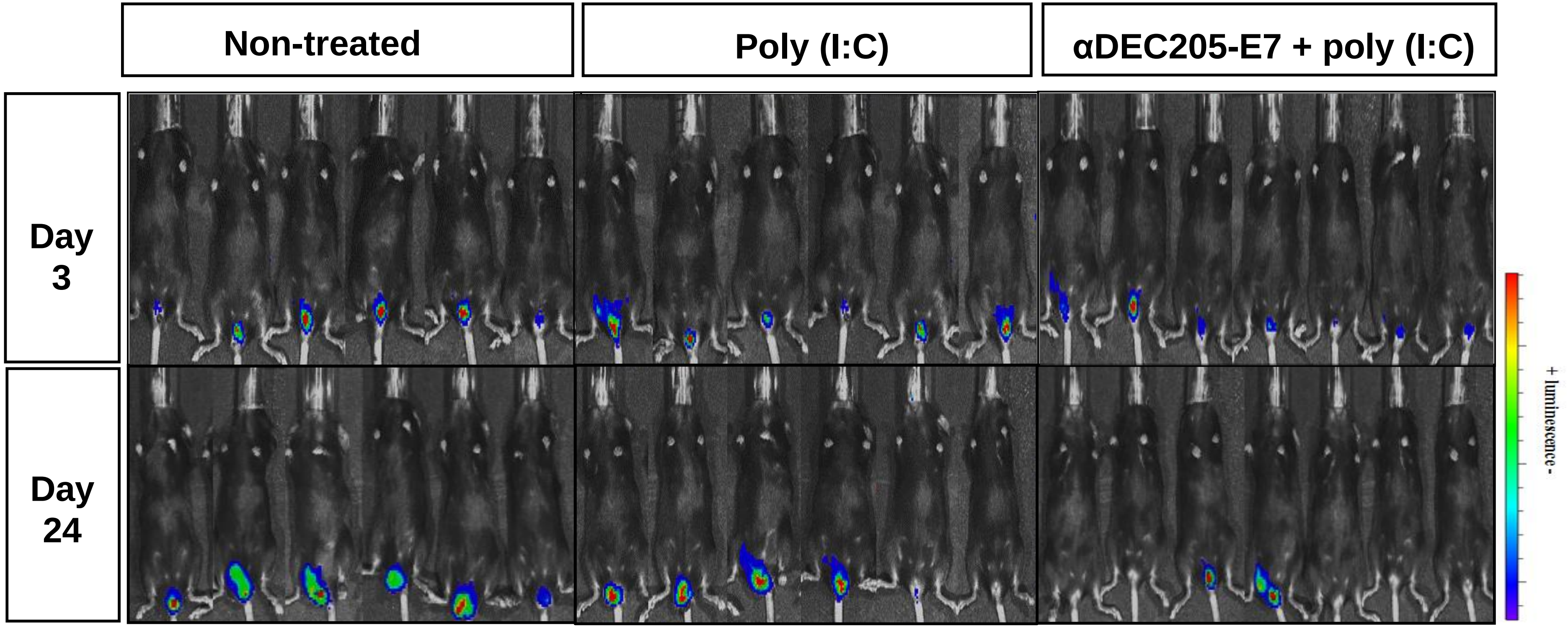

Figure S5. Immunization with the αDEC205-E7 mAb induces therapeutic antitumor effects in mice transplanted with TC-1 luc cells at in the genital mucosa. Female C57BL/6 mice received medroxyprogesterone acetate (3 mg/mouse) and, 4 days later, were engrafted with 10^5 TC-1-luc cells in the genital mucosa. Three and 10 days later, the animals were s.c immunized with 10 µg of αDEC205-E7 mAb admixed with poly (I:C). Tumor protection was evaluated once a week by bioluminescence measurement (p/sec/cm^2/sr) and images are representative from two experiments after luciferase activity 5 min after luciferin injection.

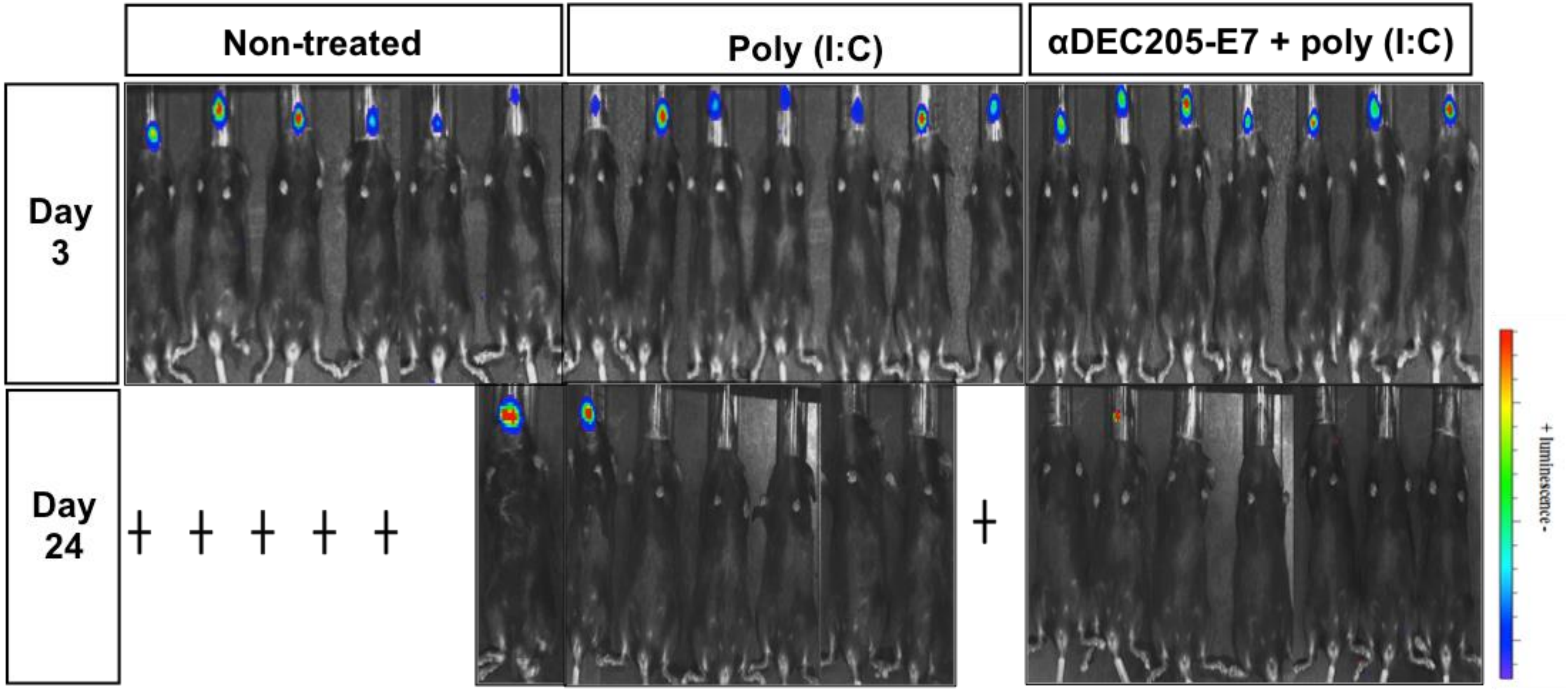

Figure S6. Immunization with the αDEC205-E7 mAb induces therapeutic antitumor effects in mice transplanted with TC-1 cells at tongue. C57BL/6 mice were engrafted with 5x10^4 TC-1-luc cells in the tongue and s.c immunized 3 and 10 days later with 10 μg of αDEC205-E7 mAb admixed with poly (I:C) or treated with only poly (I:C). Tumor protection was evaluated once a week by bioluminescence measurement p/sec/cm2/sr) and images are representative from two experiments after luciferase activity 5 min after luciferin injection.

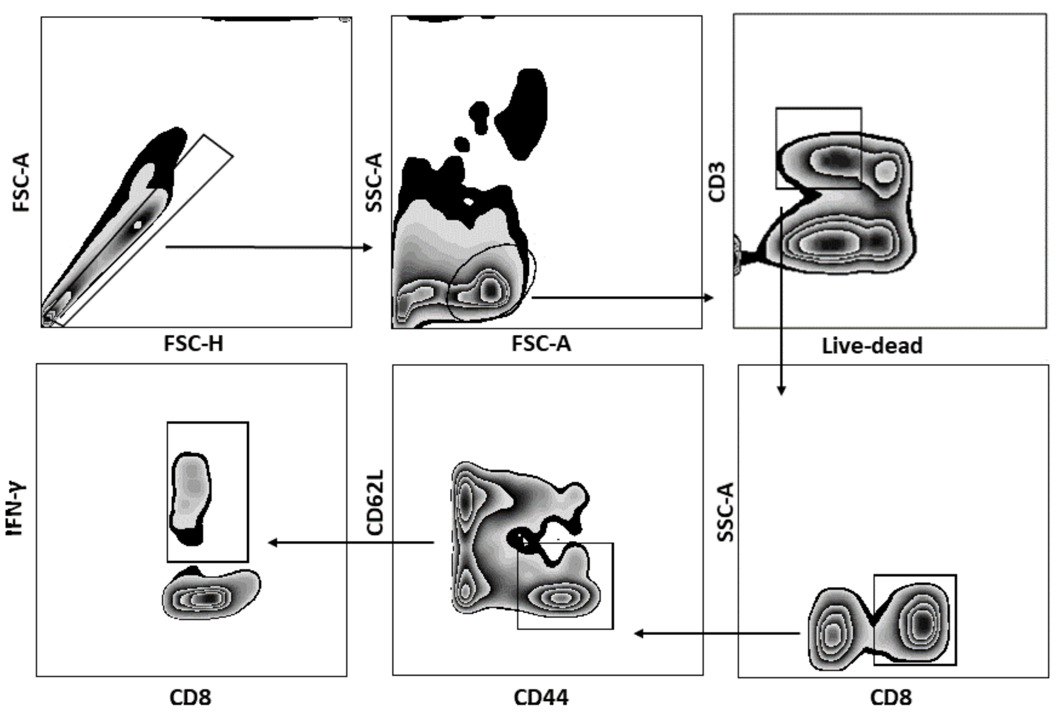

Figure S7. Gating strategy for the evaluation of IFNγ-production by effector memory CD8$^+$T cells in blood. Mice were reinjected with 5 x 10^5 TC-1-luc in the tongue as described in Figure 5. After seven days, blood cells were collected and stimulated in vitro overnight with a peptide from E7 corresponding to the K^b MHC class I–restricted epitope. The gating strategy is shown: single cells, size x granulosity, live cells$^+$ x CD3$^+$, size x CD8$^+$, CD44$^+$ x CD62L$^-$ and CD8$^+$ x IFN-γ$^+$. Analyses were performed using FlowJo software.
